# Trends in UK cancer trials: results from the UK Coordinating Committee for Cancer Research National Register of Cancer Trials

**DOI:** 10.1038/sj.bjc.6602425

**Published:** 2005-03-08

**Authors:** C Vale, L Stewart, J Tierney

**Affiliations:** 1 Meta-analysis Group, MRC Clinical Trials Unit, 222 Euston Road, London NW1 2DA, UK

**Keywords:** randomised controlled trials, trial registration

## Abstract

We aimed to study trends in the design and conduct of randomised controlled trials (RCTs) in cancer in the UK, using the UK Coordinating Committee for Cancer Research (UKCCCR) National Register of Cancer Trials (NRCT). We conducted a descriptive survey of 520 UK RCTs in cancer that were registered on the UKCCCR NRCT. All trials had been initiated between 1971 and 2000. Trials on the NRCT have been conducted in a wide variety of cancer types, but with a third in breast (22%) or lung cancer (11%). They have largely been funded by the UK public and charity sectors. Overall, there has been a sustained rise in the total numbers of patients entering UK cancer trials over time with a trend towards larger, multicentre trials, greater recruitment targets and a marked reduction in the average time taken to complete trials. Trends in the design and conduct of noncommercial cancer RCTs from 1971 to 2000 are encouraging. It will be interesting to see how they develop in light of the implementation of recent national initiatives regarding cancer clinical trials in the UK.

The recent report of the Academy of Medical Sciences (AMS) highlighted a decline in clinical research being conducted through the NHS (Bell, 2003). Furthermore, Chalmers *et al* (2003) showed that across health care, funding for noncommercial clinical trials had declined with the result that fewer trials were funded in 2002 than during the mid-1990s. There had been no accompanying increase in the planned size of these trials or the numbers of patients entering trials in this period. This is in spite of the commitments made as part of the NHS Plan (NHS Plan for England, 2000) to increase the proportion of NHS patients treated in the context of randomised controlled trials (RCTs).

However, an earlier review of published MRC-funded RCTs in solid tumours conducted between 1962 and 1995 suggested improved prospects for cancer RCTs (Machin *et al*, 1997). The authors recommended that there should be a move towards large-scale, multicentre collaborative RCTs in all types of cancer. To assess whether these recommendations had been widely realised and to explore whether the patterns identified by Chalmers *et al* (2003) were true in oncology, we investigated cancer trials assimilated on the the UK Coordinating Committee for Cancer Research (UKCCCR) National Register of Cancer Trials (NRCT) (Fayers, 1995; Fayers *et al*, 1995; Grosse *et al*, 1998). As it is a prospective trial register, the NRCT incorporates trials that failed to reach full recruitment or did not result in publication as well as those that were successfully completed. Data are therefore free from publication bias and other forms of selective reporting biases. Thus, we aimed to establish information concerning the ‘epidemiology’ of UK RCTs in cancer and to establish a baseline with which to compare future trends.

## MATERIALS AND METHODS

Since 1997, registration of UK RCTs in all types of cancer on the UKCCCR NRCT has been actively managed through the Meta-analysis Group of the MRC Clinical Trials Unit. This is achieved through contacts established across UK trials centres such that trials are registered at (or soon after) their inception. Trialists register information on each trial such that we collect a ‘core’ data set for each trial. This includes, for example, an ID number, the trial objectives, eligibility criteria, the main outcome(s), recruitment targets and contact details for the main trial personnel. This summary information can be registered either on-line (via the NRCT website) or by submitting paper registration forms or supplying a trial protocol to the register manager. The trial summaries provided are then verified prior to being made available through the NRCT website. Trial records are stored in a relational Access database and all trial protocols are kept on file. For this study, appropriate data were exported from the main register database into SPSS or Excel spreadsheets for further manipulation and analysis.

By grouping trials together in 5-year cohorts from 1971 to 2000, based on the date that patient recruitment began, we could examine whether trial characteristics had changed over time and investigate any changes that were observed. We planned to explore the numbers of trials opening overall and by disease site, by single centre or multiple centres and by funding sources. We also looked at the planned and actual recruitment of patients into these trials within these time periods and examined whether the actual accrual met the targets.

## RESULTS

### The trials

In all, 610 RCTs are registered on the NRCT. However, trials initiated between 1962 and 1970 (*n*=11) and those initiated post-2000 (*n*=22), as well as those that had been registered but had not yet begun recruitment (*n*=3) were excluded from these analyses. We also excluded any trials where data on the date recruitment started, the disease site, the funding source or whether it had been conducted at a single centre or multiple centres was missing (*n*=54). These exclusions meant that all analyses could be based on the same subset of 520 trials (85% of the total) on the register, initiated in 5-year cohorts from 1971 to 2000.

**Figure 1:**
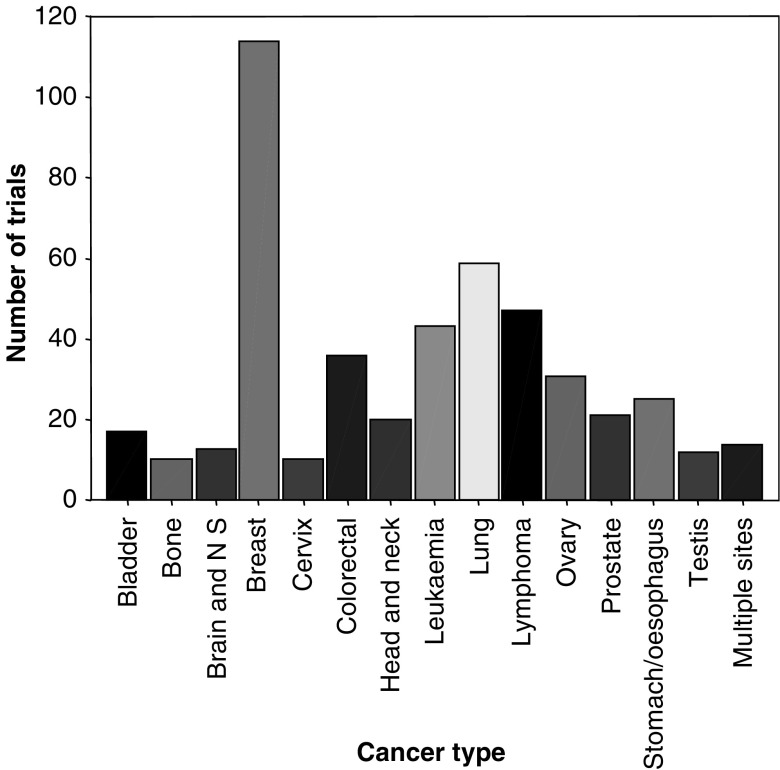
Number of trials registered per disease area.

The trials were conducted in a variety of different types of cancer (Figure 1), with breast cancer being the most frequent (114 trials), almost double that of lung cancer (59 trials), which was the next most common. Between 20 and 50 trials had looked at interventions for leukaemia, lymphoma, colorectal, ovarian, stomach and prostate cancer. In addition, a small number of trials have been conducted in a range of other cancer types including cancer of the endometrium/uterus (two trials), kidney (nine trials) and pancreas (five trials), soft tissue sarcoma (three trials) and melanoma (five trials).

**Figure 2:**
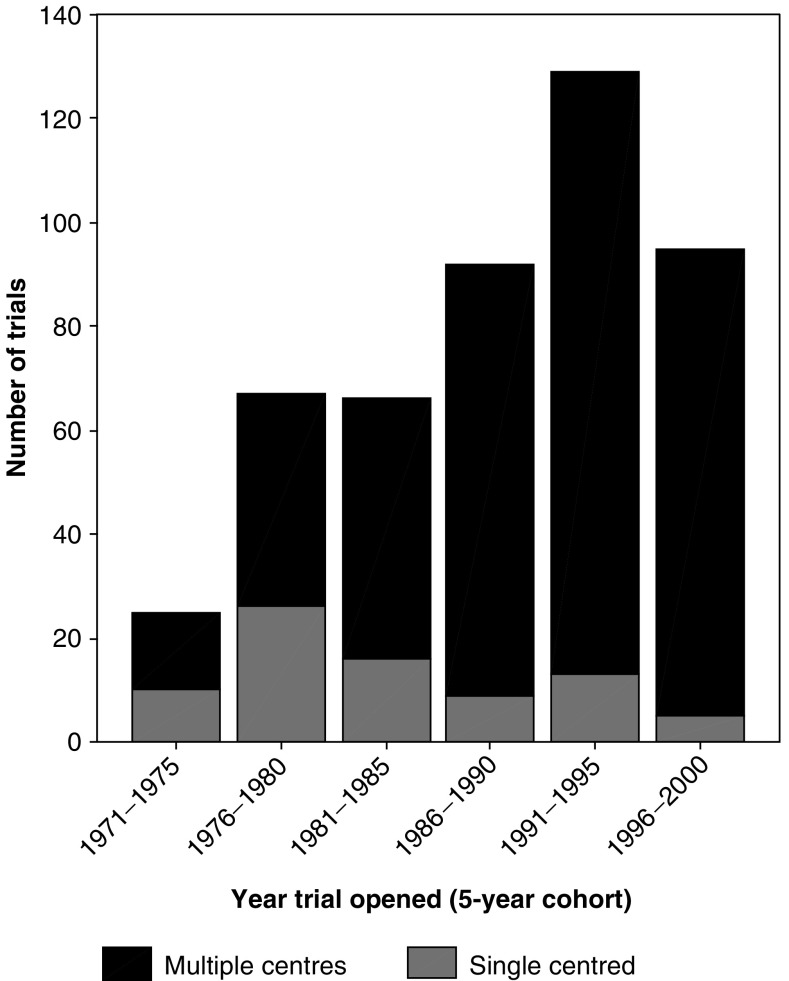
Number of trials opening per cohort (by 5-year period), showing proportions of single- and multiple-centre trials.

The largest funders of trials recorded on the UKCCCR NRCT were the UK Medical Research Council (MRC), who funded 30% of all trials, and Cancer Research UK (formerly the Cancer Research Campaign and the Imperial Cancer Research Fund), who funded a further 27%. The next largest funders were the pharmaceutical industry (17%), charities other than CRUK (14%) and the NHS/DoH (7%). The majority of the trials were multicentre (83%), and over time, the proportion of single-centre trials conducted in the UK has fallen dramatically from 40% in the 1971–1975 cohort down to only 5% in the 1996–2000 cohort (Figure 2). Figure 2 shows a steady rise in the numbers of new trials opening from the 1971–1975 cohort until 1991–1995 cohort, from 27 to 134. However, this fell to 95 new trials for the 1996–2000 cohort. Further investigation of this decline suggests that it is largely due to changes in one large trial centre in the UK (MRC Clinical Trials Unit). The number of new randomised trials conducted by this group fell from 25 in the period 1991–1995 to 10 in 1996–2000.

### Trial size and recruitment

**Figure 3:**
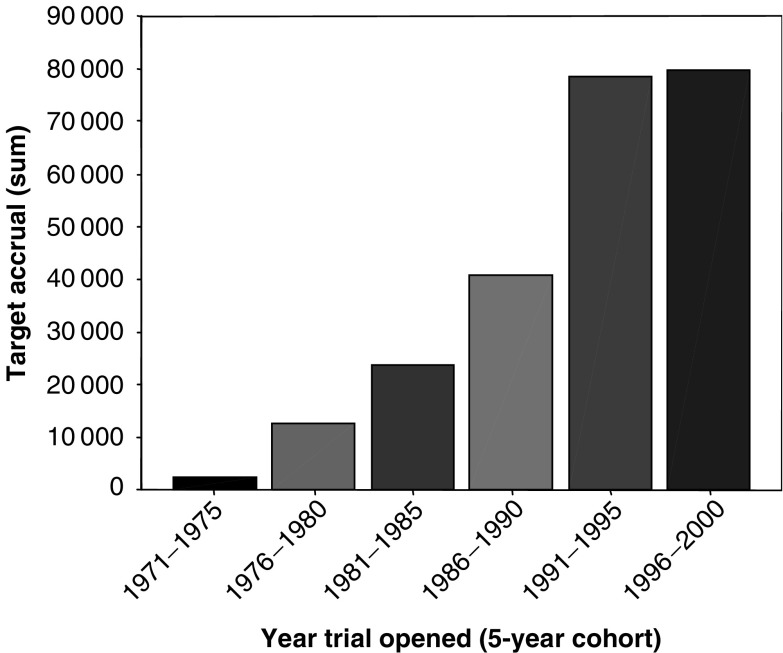
Target recruitment (sum) for trials opening within 5-year cohorts.

**Figure 4:**
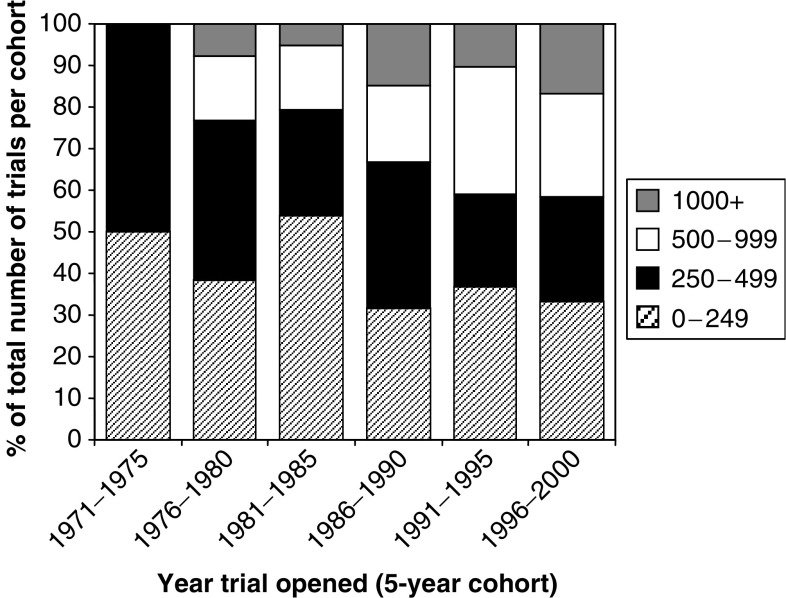
Target recruitment for trials opening within 5-year cohorts.

For these analyses, we further limited the trial subset to those where the completion dates/final number of patients (completed trials) or the current date/current number of patients (ongoing trials) were known. The resulting sample included 404 trials, 71 of which were ongoing. The median target recruitment for these 404 trials was 328 patients (range 200–460) and the sum total of target recruitment for all trials has increased steadily over time (Figure 3). Further investigation of this trend showed that it is influenced by two distinct factors: firstly, an increase in the overall number of trials conducted (Figure 2) and, secondly, an increase in the numbers of trials with the largest recruitment targets. Figure 4 shows that in the 1996–2000 cohort, more than 40% of all trials aimed to recruit 500 or more participants compared with 0% of trials in the 1971–1975 cohort.

**Figure 5:**
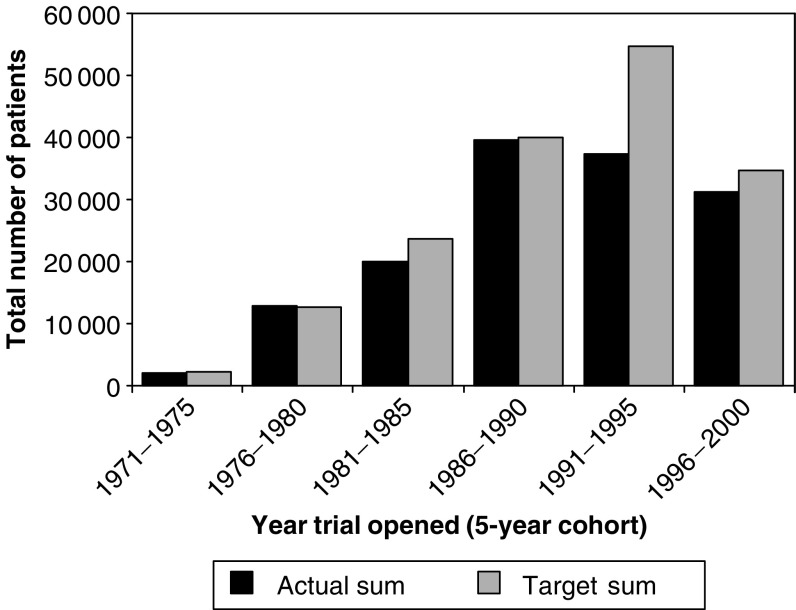
Actual and target accrual (sum) by 5-year cohort. NB. Accrual for the 1996–2000 cohort appears low as many of the larger trials in this cohort are ongoing and are therefore excluded from this analysis.

**Figure 6:**
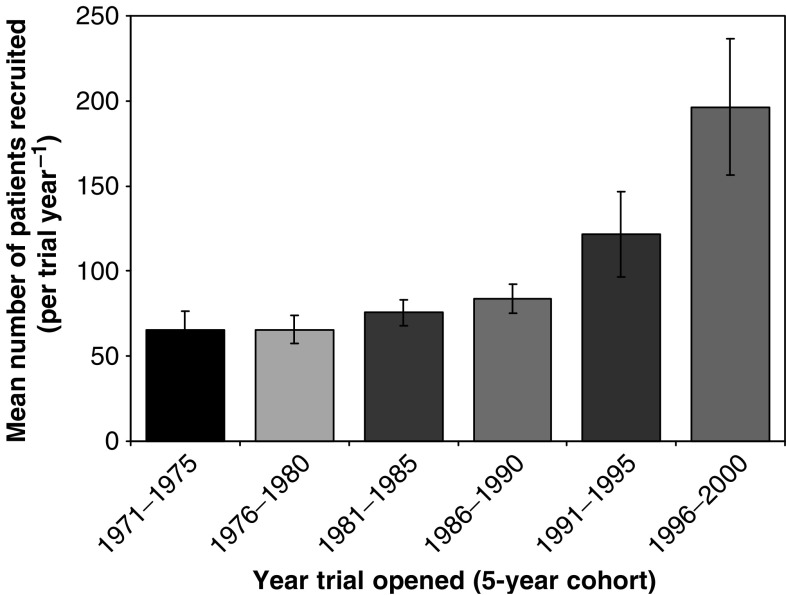
Trial recruitment rate per year (mean±s.d.) for trials opening in 5-year cohorts.

For the 333 trials that had completed recruitment, just under half (48%) reached or exceeded the planned size, 19% of all trials recruited at least 75% of the planned numbers of patients, while 20% recruited less than 25% of the planned number of patients. Figure 5 shows the relationship between the sum of the target numbers for all trials and the actual recruitment over time. It can be seen from this plot that although the target for trials that opened in the period 1991–1995 was the largest, at slightly below 55 000 patients, the actual number of patients recruited into trials in this cohort was similar to that of the previous cohort (1986–1990) at around 40 000 patients. Figure 6 shows that the mean number of patients recruited per trial per year has increased over time, from 65 patients (1971–1975) up to 197 patients (1996–2000). The impact of this increased recruitment rate has been that even with increases in trial size, the time taken to complete recruitment has fallen, such that the median duration of trials that recruited at least 90% of the target had fallen from 7.2 years (1971–1975) to 2.7 years (1996–2000).

## DISCUSSION

The UKCCCR NRCT is the longest-established prospective cancer trials register in the UK. However, there is no compulsion for trials in the UK to be routinely registered and so, although extensive, the UKCCCR NRCT is not fully comprehensive. The majority of pharmaceutical companies do not routinely register their trials and hence we currently have no way to ascertain what proportion of commercial RCTs is represented in the NRCT. There are potentially many more commercial trials in the UK than are recorded here. Similarly, although it conducts trials through centres in the UK, the European Organisation for the Research and Treatment of Cancer (EORTC) does not routinely register its trials on the UKCCCR NRCT. The NRCT should therefore only be considered representative of publicly funded and charity funded UK trials. It is also possible that the trials we do not identify and register are those being conducted in isolation in academic departments or clinics. This could have introduced some bias into these results since these are possibly the more poorly resourced, smaller-scale, single-centre trials. Furthermore, these trials may be likely to have lower rates of recruitment than those conducted through established networks of clinicians and hospitals. Nevertheless, the UKCCCR NRCT has provided a unique and useful tool to monitor trends in UK noncommercial RCTs in cancer over the last 30 years. Trials on the NRCT have been conducted in a wide variety of cancer types, although almost a third of these trials have been in either breast cancer or lung cancer. Overall, there has been a sustained rise in the total numbers of patients entering UK cancer trials over time with a trend towards larger, multicentre trials, greater recruitment targets and, importantly, a marked reduction in the average time taken to complete recruitment to trials.

Like Chalmers *et al* (2003), our findings showed that the number of new trials being initiated rose steadily from the early 1970s to a peak in the mid-1990s. However, in cancer, although fewer trials were initiated in the final cohort (1996–2000), the overall planned recruitment continued to increase. Furthermore, the average duration of trials steadily decreased as the rate of recruitment into trials rose to maximum in the 1996–2000 cohort. Since RCTs in cancer dominate the noncommercial sector, our data could imply that the true decline in other areas of health care may, in fact, be more acute than reported previously (Bell, 2003; Chalmers *et al*, 2003).

For cancer, our results show moves towards larger, multicentre collaborative group trials, possibly representative of the strong base for cancer clinical trials in the UK. They demonstrate an encouraging baseline, particularly bearing in mind that they predate current initiatives to improve the infrastructure for cancer clinical trials within the NHS, notably the formation of the National Cancer Research Institute (NCRI) and the National Cancer Research Network (NCRN). The original aim of the NCRN to double the numbers of cancer patients being treated in clinical trials by 2006 has already been achieved, suggesting that potentially more cancer patients are already being treated in the context of RCTs. The newly established UK Clinical Research Collaboration (UKCRC) aims to set up research networks in Alzheimers’ disease, diabetes, mental health, stroke and childrens’ medicine (Coombes, 2004). These should positively influence clinical research in these areas and help to reverse the trends recently identified (Bell, 2003; Chalmers *et al*, 2003).

It will be interesting and important to revisit these analyses to find out the full influence of the NCRI and NCRN initiatives on UK RCTs in cancer, and furthermore, to investigate whether recent legislative changes (European Union Directive, 2001; The medicines for human use (clinical trials) regulations, 2004) and the response to the MRC Clinical Trials for Tomorrow review (Medical Research Council, 2003) have impacted on these trends.
